# Australian health care workers experience of PPE related side-effects. A cross-sectional survey

**DOI:** 10.3389/fpubh.2024.1325376

**Published:** 2024-02-07

**Authors:** Karen McKenna, Stephane Bouchoucha, Bernice Redley, Anastasia Hutchinson

**Affiliations:** ^1^Deakin University Geelong, Faculty of Health, School of Nursing & Midwifery, Geelong, VIC, Australia; ^2^Deakin University Geelong, Institute of Health Transformation, Centre for Quality & Patient Safety Research, Geelong, VIC, Australia; ^3^Deakin University Geelong, Centre for Innovation in Infectious Diseases and Immunology Research, Geelong, VIC, Australia; ^4^Adjunct Faculty, Manipal College of Nursing (MCON), Manipal Academy of Higher Education (MAHE), Manipal, India

**Keywords:** COVID-19, personal protective equipment, pandemic, healthcare workers, side effects, pressure injuries

## Abstract

**Introduction:**

Protection of health care workers (HCWs) is a fundamental aspect of an effective pandemic response. During the COVID-19 pandemic, frequency, and duration of Personal Protective Equipment (PPE) use increased. The experience of PPE-related side-effects has potential to contribute to decreased compliance resulting in breaches in infection prevention and increasing risk of HCW exposure. This study aims were to measure the frequency of PPE-related side-effects amongst HCW in Australia, and to establish if an increased frequency of adverse reactions was related to the significant increase in use and extended duration of time spent in PPE.

**Methods:**

A descriptive cross-sectional survey was used.

**Results:**

Of the 559 respondents the majority were female (83.7%), aged 31–45 years old (33.6%). A pre-existing skin condition was reported by 266 (47.6%). Frequency of PPE related side-effects were: pressure-related 401 (71.7%), skin 321 (57.4%) and respiratory 20 (3.6%). Surgical mask use was significantly associated with pre-exiting skin conditions (*β* = 1.494 (*SE* 0.186), df (1), *p* < 0.001). Side effects to N95 respirator use was more commonly reported by staff working in COVID-19 high-risk areas (*β* = 0.572 (*SE* 0.211), df (1), *p* = 0.007) independent of work duration (*β* = −0.056 (*SE* 0.075), *df* (1), *p* = 0.456), and pre-existing skin conditions (*β* = 1.272, (*SE*.198), *df* (1), *p* < 0.001).

**Conclusion:**

The COVID-19 pandemic has seen a significant increase in the use of PPE. While the preventative benefits of PPE are significant, adverse events related to PPE use are frequently reported by HCW. Findings in this study highlight the need for innovation in PPE design to maximize protection while decreasing adverse effects and maintaining adhere to use.

## 1 Introduction

Protection of health care workers (HCWs) from exposure and infection with novel pathogens is a fundamental aspect of implementation of an effective pandemic response. In many countries high rates of HCWs infection with SARS-CoV-2 (COVID-19) have been reported to be associated with the provision of direct care to patients or residents with COVID-19 infection. This highlights inadequacies in infection prevention that may be related to supply chain disruptions or inappropriate use of Personal Protective Equipment (PPE). Factors that may influence staff adherence to PPE guidelines include workforce shortages, mental fatigue, inadequate PPE supplies, knowledge of guidelines and correct donning and doffing procedures, and the experience of PPE-related side-effects (1–4).

The Australian Government COVID-19 guidelines list HCWs as persons at increased risk of exposure (5) and the Victorian Department of Health (6) reported in 2020/2021, that 3,561 healthcare workers were infected with COVID-19, and of those cases 2,599 (73.0%) acquired the infection within the healthcare setting. Amongst HCW who had occupational acquisition 1,300 (50.0%) were aged care workers, 1,038 (39.9%) were nurses or midwives, 124 (4.8%) were medical practitioners and 83 (3.2%) were other clinical occupations. There were 609 non-clinical HCW who acquired COVID-19 in Victoria, 348 (57.1%) cases were acquired in the health care setting, of which 123 (35.3%) were cleaners and 73 (21.0%) were food services staff. These findings highlight the importance of understanding factors that may influence staff behaviors and adherence to PPE guidelines during the COVID-19 pandemic, including adverse reactions to PPE that can influence compliance with use.

Due to the increased presentation of patients with respiratory illnesses and their unknown COVID-19 status, and to prevent transmission to HCW, PPE requirements for use were increased across all areas of the Health Service, with HCW mandated to wear PPE at all times (7). PPE use was extended from providing care to patients on transmission-based precautions, when transitioning between patients, and when in public areas of the hospital where social distancing could not be achieved. This practice was new for some staff in some clinical areas, with many reporting they were unfamiliar with practices associated with donning and doffing PPE, with the exception of HCWs employed the operating suite, or those in clinical areas caring for patients on droplet or airborne transmission-based precautions. The significance of the increased use of PPE can be seen from the analysis of PPE consumption data, demonstrating that there was an exponential increase in PPE supply and usage. In a 6–12 month period during the pandemic, the use of P2/N95 masks was equivalent to 25 years of pre-pandemic use, and the storage of long-sleeve gowns increased significantly, with a reported 40,000 gowns maintained on-site as a reserve supply (8). Guidance provided to HCWs during the COVID-19 pandemic on the minimum PPE standard for providing care to patients with suspected or confirmed COVID-19 included: respiratory protection in the form of surgical masks and P2/N95 respirators, long sleeve gowns, eye protection including glasses and face shields, and gloves (9). Hand hygiene practices were also considered to be fundamentally important during the pandemic and included the use of soap and water, and alcohol-based hand rub (ABHR) formulations (10). Irritation and allergic reactions have been reported from the use of hand hygiene products (11), and a higher incidence of hand dermatitis was associated with the frequency of hand hygiene undertaken (12).

In addition, access to PPE at the beginning of the pandemic was challenging, due to a global surge in demand and subsequent PPE supply shortages (2). This meant that in some health care settings HCW were required to wear PPE continuously throughout their shift, increasing their risk of experiencing PPE-related side-effects (11, 13). These factors all have the potential to contribute to decreased compliance with PPE guidelines, increasing the risk of HCW exposure to COVID-19 infection.

The presence of adverse facial reactions to PPE has been reported to be higher for female HCWs (7, 14). And being a female nurse has been identified as a risk factor for adverse dermatological reactions associated with PPE during the COVID-19 pandemic (15). The Australian health workforce is predominantly made up of female employees, with the health workforce data reporting the makeup of the workforce in 2021 as 88.4% of the nursing/midwifery workforce were female, and 43.9% of the medical practitioner workforce were female (16).

The increased use of PPE, and duration of time spent in PPE was reported across the world during the pandemic, with some studies reporting a higher incidence of PPE-related complications in HCW groups caring for patients with COVID-19 (12). It has also been reported that HCW experiencing exacerbation of pre-existing skin conditions and skin breakdown attempted to manipulate the contact points of the PPE to reduce pressure, thereby reducing its effectiveness (12). Prolonged use of PPE has also been associated with the exacerbation of pre-existing skin conditions, including rosacea and acne, caused by the humid environment created by the use of surgical and P2/N95 masks, and the tight seal from P2/N95 masks applying pressure to the skin causing the breakdown of skin, rupture of lesions, and increase in the prevalence of bacteria (7).

This study aims were to measure the frequency of PPE-related side-effects amongst HCWs in Australia, related to the increased frequency of use and extended duration of time spent in PPE. The following aspects of PPE use were measured: (i) physical side effects of PPE from pressure issues, occurrence of skin abrasion and breakdown, or worsening of underlying skin conditions, (ii) increased infection risks associated with the breakdown of the skin barrier, (iii) staff wearing PPE incorrectly PPE to improve comfort and thereby reducing its effectiveness: e.g., wearing a surgical mask in place of a N95 mask.

## 2 Methods

### 2.1 Study design

A cross-sectional survey was used to explore the experience of both clinical and non-clinical HCWs’ use and side-effects associated with PPE in 2020–21.

### 2.2 Study site

This study took place in a large health service provider that offers a unique perspective as it includes a network of hospitals and provides care to the population in both the public and private sectors, and across primary, residential aged care, subacute ambulatory care, and acute health service delivery.

The results of the survey are summarised and presented according to site within the health service. The study sites have been de-identified and categorized as site A, B and C for the purpose of data analysis. With the metropolitan hospital that provides specialist women’s and children’s acute care (A), a second metropolitan hospital that provides general acute, subacute, and mental health services (B). And a third smaller rural health service that provides sub-acute care and multiple residential aged care facilities across 4 states (C).

Throughout the pandemic, the State Department of Health PPE guidelines were used to provide the recommendations for the minimum level of PPE for low, medium, and high-risk settings (9). At the commencement of the survey the minimum level of PPE required by all employees was a surgical mask with all employees in clinical and patient facing areas required to wear eye protection and a surgical mask. N95 respirators were required in high-risk areas (Emergency Department, ICU and COVID-19 wards), and Tier-3 PPE (N95 respirator, eye protection, long sleeve gown and gloves) was required when caring for patients with suspected or confirmed COVID-19 infection (9).

### 2.3 Sample and recruitment

Staff were recruited through advertising via flyers circulated in staff break-out areas, email and the health service internal social media platform ‘Workplace’. The survey was made available electronically and the link was distributed to all clinical staff working within the network. The survey was active between December 2020 and November 2021.

Inclusion criteria were front-line HCWs (nurses, midwives, medical and allied health staff) and non-clinical HCWs (cleaners, ward clerks) working. Exclusion criteria were individuals who did not provide informed consent by completing the online survey, staff members less than 18 years of age, and staff who were not permanent employees of the health service.

### 2.4 Data collection tools

The survey data collection tool was developed with an expert panel of international Infection prevention leaders and was informed by a rapid review of the current literature (3). The questions were based on issues related to PPE that were topical throughout the pandemic including: types and varieties of PPE used, side-effects and injuries sustained, staff knowledge. The survey was tested with ten staff to confirm readability and clarity prior to distribution. External validity was evaluated by comparing the survey responses to those obtained by other research groups using the tool in Singapore (17).

The survey tool included information on participants age, sex, occupation, employment status and work location, and PPE usage patterns and associated side-effects. Outcomes were measured using a mix of yes/no questions, open ended questions, and a Likert scale with responses ranging from strongly disagree (1) to strongly agree (5), and rating responses on a scale of 1–10 with 1 being very poor and 10 excellent.

### 2.5 Sample size and survey analysis

Assuming that the true prevalence of PPE-related side-effects is 25.0% and that the survey instrument has a sensitivity of 70.0% and a specificity of 70.0%, a sample size of 550 participants is sufficient to detect the true prevalence of side-effects +/−10%. In addition, observational studies using logistic regression need to have a minimum sample size of 500 cases for the derived outcomes to represent the underlying characteristics of the study population (18, 19).

Data were downloaded from Qualtrics, cleaned and analysed using SPSS 28®. As the survey was distributed electronically and/or accessed via a QR Code, response rates were calculated as the total number of completed surveys divided by the total number of surveys accessed by potential participants. Work locations were classified into high, medium and low risk, based on the health service work health and safety risk classification and Respiratory Protection Program risk matrix (20).

Data were summarised using descriptive statistics. Logistic regression was used to analyze the association between the experience of side-effects associated with surgical mask use and N95 mask use after adjusting for area of clinical work (high-risk of COVID-19 exposure versus low or medium), the existence of pre-exiting skin conditions and the average number of hours worked.

### 2.6 Ethics statement

Ethics approvals were obtained from the institutional Human Research Ethics Committee (HREC) approval number: 2021–014 and the university. Participation in the study was anonymous and voluntary. Informed consent was implied by participants completing the survey.

## 3 Results

There were 627 respondents with 559 surveys who completed at least one section of the survey and a survey completion rate of 89.2%. Most participants were female (83.7%) aged between 31–45 years old (33.6%), (Table 1). Most respondents were nurses/midwives (66.0%), followed by allied health (5.0%), and 87 (15.6%) did not specify their occupation. A pre-existing skin condition was reported by 266 (47.6%) participants, with dry skin most frequently reported (28.8%) followed by atopic dermatitis (19.0%) and allergic dermatitis (9.5%).

**Table 1:** Participant Demographics.

	A *n* = 161 *n* (%)	B *n* = 350 *n* (%)	C *n* = 48 *n* (%)	Total *n* = 559 *n* (%)
Gender				
Female	151 (93.8)	281 (80.5)	36 (75.0)	468 (83.7)
Male	9 (5.6)	64 (18.3)	11 (22.9)	84 (15.0)
Non-binary	0	3 (0.9)	0	3 (0.5)
Not specified	1 (0.6)	2 (0.6)	1 (2.1)	4 (0.7)
Age				
<21–30	56 (34.8)	106 (30.3)	10 (20.8)	172 (30.8)
31–45	43 (26.7)	134 (38.3)	11 (22.9)	188 (33.6)
46–60	47 (29.2)	93 (26.6)	20 (41.7)	160 (28.6)
≥ 61	15 (9.3)	17 (4.9)	6 (12.5)	38 (6.8)
Missing	0	0	1 (2.1)	1 (0.2)
Occupation				
Medical	**18 (11.2)**	**26 (7.4)**	**0**	**44 (7.9)**
Nursing / Midwifery	**112 (69.6)**	**239 (68.3)**	**18 (37.5)**	**369 (66.0)**
Registered nurse	74 (46.0)	191 (54.6)	11 (22.9)	276 (49.4)
Enrolled nurse	0	6 (1.7)	7 (14.6)	13 (2.3)
Registered midwife	37 (23.0)	35 (10.0)	0	72 (12.9)
RUSON/M	1 (0.6)	7 (2.0)	0	8 (1.4)
Allied Health	**3 (1.9)**	**23 (6.6)**	**2 (4.2)**	**28 (5.0)**
Other - clinical	**6 (3.7)**	**3 (0.9)**	**0**	**9 (1.6)**
Other - non-clinical	**5 (3.1)**	**14 (4.0)**	**2 (4.2)**	**21 (3.8)**
Not specified	**17 (10.6)**	**45 (12.9)**	**26 (54.2)**	**88 (15.8)**
Pre-existing skin conditions				
Yes	95 (59.0)	156 (44.6)	15 (31.3)	266 (47.6)
Skin conditions				
Atopic dermatitis	43 (26.7)	57 (16.3)	6 (12.5)	106 (19.0)
Allergic dermatitis	17 (10.6)	34 (9.7)	2 (4.2)	53 (9.5)
Heat rash	13 (8.1)	18 (5.1)	5 (10.4)	36 (6.4)
Dermatosis	5 (3.1)	11 (3.1)	1 (2.1)	17 (3.0)
Psoriasis	9 (5.6)	19 (5.4)	1 (2.1)	29 (5.2)
Dry skin	69 (42.9)	87 (24.9)	5 (10.4)	161 (28.8)
Acne	5 (3.1)	5 (1.4)	2 (4.2)	12 (2.1)
Rosacea	5 (3.1)	1 (0.3)	0	6 (1.1)
Urticaria	1 (0.6)	2 (0.6)	0	3 (0.5)
Others	3 (1.9)	4 (1.1)	0	7 (1.3)

RACF - Residential aged care facility. RUSON/M - Registered Undergraduate Student of Nursing/Midwifery.

Other clinical included: patient services assistant (PSA), ambulance and pathology, and non-clinical included: administration, security, food services, environmental services, information technology (IT), engineering, and instrument technician.

Most respondents worked in general acute care (30.1%), followed by critical care (26.3%) and residential aged care and sub-acute care settings (21.6%). The majority (71.6%) identified as working in a high-risk COVID-19 environment and most worked part-time (81.2%) reported working part time during the pandemic, 46.2% reported working 31 to 40 h per week, and 17.7% reported working 21 to 30 h per week (Table 2).

**Table 2:** Employment status and locations.

	A *n* = 161 *n* (%)	B *n* = 350 *n* (%)	C *n* = 48 *n* (%)	Total *n* = 559 *n* (%)
Employment status				
Full time	34 (21.1)	53 (15.1)	11 (22.9)	98 (17.5)
Part time	126 (78.3)	292 (83.4)	36 (75.0)	454 (81.2)
Casual	0	2 (0.6)	0	2 (0.4)
Missing	1 (0.6)	3 (0.9)	1 (2.1)	5 (0.9)
COVID-19 risk setting				
Low	6 (3.7)	7 (2.0)	0	13 (2.3)
Medium	36 (22.4)	69 (19.7)	5 (10.4)	110 (19.7)
High	110 (68.3)	247 (70.6)	43 (89.6)	400 (71.6)
Missing	9 (5.6)	27 (7.7)	0	36 (6.4)
Primary work location				
Critical care	**55 (34.2)**	**92 (26.3)**	**0**	**147 (26.3)**
Emergency department	19 (11.8)	52 (14.9)	0	71 (12.7)
Critical care adult	6 (3.7)	29 (8.3)	0	35 (6.3)
Critical care paediatrics	2 (1.2)	5 (1.4)	0	7 (1.3)
Critical care neonatal	31 (19.3)	12 (3.4)	0	43 (7.7)
Ambulance	0	1 (0.3)	0	1 (0.2)
Obstetrics	**51 (31.7)**	**43 (12.3)**	**0**	**94 (16.8)**
Antenatal	26 (16.1)	11 (3.1)	0	37 (6.6)
Birth Suite	32 (19.9)	38 (10.9)	0	70 (12.5)
Postnatal	34 (21.1)	31 (8.9)	0	65 (11.6)
Obstetrics community	2 (1.2)	5 (1.4)	0	7 (1.3)
Perioperative suite	**21 (13.0)**	**21 (6.0)**	**0**	**42 (7.5)**
Pre-admission clinic	2 (1.2)	4 (1.1)	0	6 (1.1)
Anesthetics	6 (3.7)	9 (2.6)	0	15 (2.7)
Operating room	15 (9.3)	14 (4.0)	0	29 (5.2)
PACU	8 (5.0)	8 (2.3)	0	16 (2.9)
Endoscopy	0	2 (0.6)	0	2 (0.4)
Acute Care	**40 (24.8)**	**123 (35.1)**	**5 (10.4)**	**168 (30.1)**
Screeners	6 (3.7)	18 (5.1)	2 (4.2)	26 (4.7)
COVID ward	2 (1.2)	24 (6.9)	1 (2.1)	27 (4.8)
Medical adult	8 (5.0)	70 (20)	2 (4.2)	80 (14.3)
Medical pediatrics	4 (2.5)	21 (6.0)	0	25 (4.5)
Surgical ward	23 (14.3)	31 (8.9)	0	54 (9.7)
Same day admission	2 (1.2)	7 (2.0)	0	9 (1.6)
RAC and Sub-acute	**17 (10.6)**	**61 (17.4)**	**43 (89.6)**	**121 (21.6)**
Palliative care	0	11 (3.1)	1 (2.1)	12 (2.1)
Mental health	0	10 (2.9)	0	10 (1.8)
HIP/HITH	0	11 (3.2)	1 (2.1)	12 (2.1)
GEM	0	2 (0.6)	2 (4.2)	4 (0.7)
RAC	1 (0.6)	4 (1.1)	40 (83.3)	45 (8.1)
Hotel quarantine	0	1 (0.3)	0	1 (0.2)
Outpatients	16 (9.9)	25 (7.1)	0	41 (7.3)
Other	**18 (11.2)**	**41 (11.7)**	**1 (2.1)**	**60 (10.7)**
Other - clinical	12 (7.5)	13 (3.7)	0	25 (4.5)
Other - non-clinical	6 (3.7)	42 (12.0)	2 (4.2)	50 (8.9)

SCN - Special Care Nursery, PACU – Post Anesthetic Care Unit, HIP Health Independence Program, GEM – Geriatric Evaluation and Management, RAC – Residential Aged Care, HITH – Hospital in The Home. COVID risk settings were determined based on the Work Health Safety (WHS) risk classification.

### 3.1 Types of PPE used

When respondents were asked about their PPE use during the pandemic (Table 3), 486 (86.9%) reported wearing a surgical mask, 392 (70.1%) reported wearing an N95 respirator and eye protection was used by 532 (95.2%).

**Table 3:** Types of PPE used during the COVID-19 pandemic.

Types of PPE used	A *n* = 161 *n* (%)	B *n* = 350 *n* (%)	C *n* = 48 *n* (%)	Total *n* = 559 *n* (%)
Types of PPE used				
Surgical mask	**138 (85.7)**	**306 (87.4)**	**42 (87.5)**	**486 (86.9)**
N95 respirator	**125 (77.6)**	**250 (71.4)**	**17 (35.4)**	**392 (70.1)**
PAPR	**0**	**3 (0.9)**	**0**	**3 (0.5)**
Eye Protection	**157 (97.5)**	**333 (95.1)**	**42 (87.5)**	**532 (95.2)**
Goggles	150 (93.2)	318 (90.9)	36 (75.0)	504 (90.2)
Face shield	97 (60.2)	234 (66.9)	29 (60.4)	360 (64.4)
Apron	40 (24.8)	78 (22.3)	11 (22.9)	129 (23.1)
Long sleeve gown	94 (58.4)	219 (62.6)	18 (37.5)	331 (59.2)
Single gloves	112 (69.6)	259 (74.0)	35 (72.9)	406 (72.6)
Double gloves	49 (30.4)	70 (20.0)	6 (12.5)	125 (22.4)
Shoe covers	38 (23.6)	76 (21.7)	10 (20.8)	124 (22.2)
Hair cover	53 (32.9)	125 (35.7)	8 (16.7)	186 (33.3)
Other	3 (1.9)	4 (1.1)	0	7 (1.3)

Powered air purifying respirators (PAPR) were not provided to staff by the organization.

### 3.2 Reported side effects from PPE use

Reported side effects experienced from PPE use are shown in Table 4. Surgical masks were commonly associated (N = 221, 39.5%) with side-effects such as: acne (21.8%), pressure injuries (18.1%) and burning/pain (8.1%) on the cheeks (22.0%), nose (19.5%) and behind the ear (16.5%).

**Table 4:** Frequency of reported side-effects by Personal Protective Equipment type (N = 559).

	Goggles	Face shield	Surgical mask	N95 respirator	No side-effects
Skin related	36 (6.4)	17 (3.0)	165 (29.5)	103 (18.4)	238 (60.5)
Skin tear	7 (1.3)	0	13 (2.3)	14 (2.5)	
Blister	7 (1.3)	1 (0.2)	18 (3.2)	16 (2.9)	
Acne	18 (3.2)	7 (1.3)	122 (21.8)	69 (12.3)	
Abrasion	8 (1.4)	4 (0.7)	44 (7.9)	31 (5.5)	
Eczema	3 (0.5)	5 (0.9)	30 (5.4)	12 (2.1)	
Allergic reaction	3 (0.5)	4 (0.7)	37 (6.6)	13 (2.3)	
Dry Skin	0	0	3 (0.5)	0	
Pressure related	**104 (18.6)**	**30 (5.3)**	**126 (22.5)**	**141 (25.2)**	**158 (28.3)**
Burning / Pain	35 (6.3)	12 (2.1)	55 (9.9)	53 (9.5)	
Pressure injuries	57 (10.2)	16 (2.9)	101 (18.1)	124 (22.2)	
Headache / migraine	32 (5.7)	10 (1.8)	7 (1.3)	6 (1.1)	
Vision	**25 (4.5)**	**16 (2.9)**	**0**	**0**	
Blurred vision	15 (2.7)	4 (0.7)	0	0	
Impaired vision	2 (0.4)	10 (1.8)	0	0	
Fogging	9 (1.6)	5 (0.9)	0	0	
Hearing	**0**	**3 (0.5)**	**0**	**0**	
Impaired hearing	0	3 (0.5)	0	0	
Respiratory	**0**	**2 (0.4)**	**9 (1.6)**	**9 (1.6)**	
Asthma	0	0	3 (0.5)	1 (0.2)	
Shortness of breath	0	0	0	5 (0.9)	
Respiratory - other	0	2 (0.4)	6 (1.1)	3 (0.5)	
Others	**1 (0.2)**	**1 (0.2)**	**10 (1.8)**	**5 (0.9)**	

*Participants could report side-effects associated with multiple types of PPE.

In relation to N95 respirator use, 30.4% reported an adverse effect with pressure injuries the most common (22.2%) followed by acne (12.3%). The location of side effects was similar to those reported with surgical masks, with nose (23.6%), cheeks (17.2%) and behind the ear (9.5%) the most frequently reported. The frequency of side-effects according to the hospital site is provided in Appendix 1.

The frequency of side effects reported increased proportionally with the number of hours worked per week (Table 5). Despite this, logistic regression analysis found that individuals who worked in areas where there was high risk of caring for patients with known of suspected COVID-19 infection were more likely to report side-effects associated with the use of N95 respirators (*β* = 0.572 (*SE* 0.211), df (1), *p* = 0.007) independent of the number of hours worked (*β* = −0.056 (*SE* 0.075), *df* (1), *p* = 0.456), after adjusting for the presence of pre-existing skin conditions (*β* = 1.272, (*SE* 0.198), *df* (1), *p* < 0.001). In contrast the experience of side-effects associated with surgical mask use was significantly associated with the existence of pre-exiting skin conditions (*β* = 1.494 (*SE* 0.186), df (1), *p* < 0.001) independent of the clinical area of work (*β* = −0.119 (*SE* 0.209), df (1), *p* = 0.567) or the number of hours worked per week (*β* = − 0.016 (*SE* 0.073), df (1), *p* = 0.828).

**Table 5:** Reported side-effects of PPE and number of hours worked per week.

Hours worked	Skin side-effects *n* (%)	Pressure side-effects *n* (%)	Respiratory side-effects *n* (%)
1–20	33 (10.3)	49 (12.2)	1 (5.0)
21–30	56 (17.5)	56 (14.0)	3 (15.0)
31–40	153 (47.7)	176 (43.9)	12 (60.0)
Not specified	79 (24.6)	120 (29.9)	4 (20.0)
Total	**321 (57.4%)**	**401 (71.7%)**	**20 (3.6%)**

### 3.3 Hand hygiene practices

Approximately a third (197, 35.2%) of respondents reported an adverse reaction to hand hygiene products (Table 6). The most reported were skin dryness (34.2%), followed by scaly skin (18.2%) and itch (16.3%). Skin reaction severity was reported as varying between slight to moderate.

**Table 6:** Hand hygiene practices during the COVID-19 pandemic.

	A *n* = 161 *n* (%)	B *n* = 350 *n* (%)	C *n* = 48 *n* (%)	Total *n* = 559 *n* (%)
Method of hand hygiene				
Hand washing	137 (85.1)	307 (87.7)	40 (83.3)	484 (86.6)
ABHR	152 (94.4)	317 (90.6)	42 (87.5)	511 (91.4)
Other	3 (1.9)	0	0	3 (0.5)
Frequency of hand washing per shift				
0–10	113 (70.2)	203 (58.0)	18 (37.5)	334 (59.7)
11 20	15 (9.3)	40 (11.4)	14 (29.2)	69 (12.3)
21–40	7 (4.3)	21 (6.0)	0	28 (5.0)
41–50	2 (1.2)	14 (4.0)	3 (6.3)	19 (3.4)
>50	1 (0.6)	11 (3.1)	2 (4.2)	14 (2.5)
Not specified	21 (13.0)	61 (17.4)	11 (23)	93 (16.7)
Frequency of ABHR per shift				
0–10	19 (11.8)	49 (14.0)	11 (22.9)	79 (14.1)
11–20	36 (22.4)	69 (19.7)	11 (22.9)	116 (20.8)
21–40	26 (16.1)	72 (20.6)	9 (18.8)	107 (19.1)
41–50	18 (11.2)	46 (13.1)	4 (8.3)	68 (12.2)
>50	42 (26.1)	53 (15.1)	3 (6.3)	98 (17.5)
Not specified	20 (12.4)	61 (17.4)	10 (20.9)	91 (16.3)
Experienced adverse effect relating to hand hygiene	62 (38.5)	118 (33.7)	17 (35.4)	197 (35.2)
Adverse effect				
Tenderness	13 (8.1)	24 (6.9)	5 (10.4)	42 (7.5)
Scaly skin	31 (19.3)	62 (17.7)	9 (18.8)	102 (18.2)
Burning / pain	18 (11.2)	17 (4.9)	3 (6.3)	38 (6.8)
Itch	29 (18.0)	55 (15.7)	7 (14.6)	91 (16.3)
Dryness	62 (38.5)	112 (32.0)	17 (35.4)	191 (34.2)
Rash	16 (9.9)	34 (9.7)	5 (10.4)	55 (9.8)
Lesions	6 (3.7)	7 (2.0)	1 (2.1)	14 (2.5)
Broken skin	2 (1.2)	2 (0.6)	0	4 (0.7)
Other	7 (4.3)	3 (0.9)	0	10 (1.8)
Severity of adverse reactions				
(slight) 1	0	2 (0.6)	0	2 (0.4)
2	8 (5.0)	16 (4.6)	0	24 (4.3)
3	11 (6.8)	25 (7.1)	2 (4.2)	38 (6.8)
4	7 (4.3)	14 (4.0)	1 (2.1)	22 (3.9)
(moderate) 5	17 (10.6)	21 (6.0)	4 (8.3)	42 (7.5)
6	5 (3.1)	12 (3.4)	3 (6.3)	20 (3.6)
7	6 (3.7)	8 (2.3)	3 (6.3)	17 (3.0)
8	2 (1.2)	4 (1.1)	0	6 (1.1)
9	1 (0.6)	1 (0.3)	1 (2.1)	3 (0.5)
(very severe) 10	0	0	0	0

## 4 Discussion

The COVID-19 pandemic has provided unique opportunities to explore the challenges faced by HCW in caring for patients with infectious diseases on a large scale through COVID-19, including side-effects of working in PPE for extended period. This study identified that PPE related side-effects were common across all areas of the organization with 71.7% of participants reporting adverse pressure-related symptoms, and 57.4% skin side-effects. The most reported reactions were to surgical masks, followed by N95 respirators. Of note, self-report of a pre-existing skin conditions increased the likelihood that staff would report side-effects associated with the use of surgical masks which may explain why surgical mask related side-effects were common amongst staff in both clinical and non-clinical roles. While side-effects associated while N95 respirator use was predominate amongst clinical staff working in high-risk areas. Similar to results in a previous systematic review (21) side-effects associated with hand hygiene practices during the COVID-19 pandemic included: skin dryness (27.6%), followed by erythema (22.1%) and contact dermatitis (14.8%) as the most frequently reported types of dermatosis.

Several studies, predominantly undertaken in Europe and Asia, have reported side effects experienced by HCW due to prolonged use of PPE during the COVID-19 pandemic (17, 22). A comparison study using the same questionnaire at a hospital in Singapore was completed in 2020 (17). Both studies reported similar participant demographics with majority of the respondents being female nurses, aged below 40 years, followed by medical and allied health staff (17). Reports of pre-existing skin conditions were similar with 45.4% of participants in the Singapore study reporting a pre-existing condition, and 47.6% in this study. The most frequently reported pre-existing conditions in the Singapore study was dry skin (58.0%), followed by atopic dermatitis (34.2%), whereas in this study, dry skin was reported most frequently by 28.8% of participants, followed by atopic dermatitis at 19.0%.

The Singapore study identified the most commonly reported symptoms associated with PPE use were related to N95 respirators, with adverse effects experienced on the nose (55.0%) and cheeks (53.0%) (17). The finding that surgical masks were most widely reported as causing side-effects is in line with the timing of survey distribution, as at this time surgical mask use (rather than N95 use) was mandated for all employees working in the state of Victoria, Australia (9). While this study reported fewer side effects related to N95 use, the frequency of location of side effects were the same as the Singapore study, with 36.6% of injuries reported on the nose, followed by 32.3% on the cheeks. This study identified that the more hours worked by HCW saw an increase in the number of reported skin reactions. Multivariate logistic regression analysis demonstrated that N95 respirator associated side-effects were associated with working in a COVID-19 high risk clinical area and the existence of pre-existing skin conditions and were independent of the number of hours worked per week. In contrast, the comparison Singapore study identified that a longer shift duration saw an increase in reported PPE associated side effects (17). An additional systematic review identified that a longer exposure duration of PPE demonstrated a positive association with adverse skin reactions that was statistically significant (21, 22). These difference in the association between the number of hours worked and N95 respirator side-effects may be influenced by the local climate (subtropical versus temperate) and other local factors.

While the requirements to use PPE as a preventative measure during the pandemic were unavoidable, the side effects from PPE use contribute to the increased burden on the overall wellbeing of HCWs. In addition to the stress associated with the risks of acquiring COVID-19 in the workplace, HCWs reported additional stressors associated with the increased duration of PPE use, including headaches, breathing difficulties and impaired cognition (7). Perceived barriers to PPE access could have a detrimental effect on correct usage of PPE, leading to increased HCW exposure to COVID-19, and a depletion of the workforce. PPE use, including the prolonged use, was new to some HCWs, with many being unfamiliar with donning and doffing processes. Staff within the operating suite were the only department in the Health Service to consistently wear surgical masks and eye-protection pre-pandemic. PPE was used periodically in other departments throughout the health service, when caring for patients in transmission-based precautions however, the time spend in PPE would be limited to patient interactions and was not consistent with the prolonged duration of PPE use during the COVID-19 pandemic (8). A study into factors that influence compliance with PPE use, identified that poor compliance and inconsistent use of PPE has been the main cause of healthcare associated transmission of COVID-19 (23). A cross-sectional survey into the physical and stressful psychological impacts of prolonged PPE use during the pandemic (24), estimated the average PPE use time as 6.8 h per shift, and describes the long hours spent in PPE as uncomfortable, with increased reports of physical, respiratory and musculoskeletal disorders related to PPE (24). Other reported barriers to effective and appropriate PPE use include, discomfort from face masks or face shield use (25). A scoping review on the implementation of PPE in healthcare (2) reported similar findings on the disruptive effect PPE use has on clinical work flow, including the time-consuming nature of donning and doffing, discomfort from overheating, breathing difficulties, and topical allergies and skin reactions to PPE that were exacerbated with extended use (2).

Incorrect PPE use has been associated with a reduction in the effectiveness of the PPE and increases the risk of transmission of COVID-19 (26). During the early stages of the COVID-19 pandemic PPE supply chains were severely impacted resulting in PPE shortages, and limited choice in the available types of PPE products, meaning HCWs were limited in their choice of P2/N95 mask and not able to access one that provided a correct fit, potentially increasing the risk of adverse reactions experienced (27). Rationing of PPE supplies that are normally indicated for single use, resulting in inappropriate or sub-optimal use may pose an increased threat and be a contributing factor to adverse reactions (7). During the COVID-19 pandemic, Elston (12) reported that attempted manipulation of the contact points of PPE to reduce pressure related to dermal reactions, resulting in the incorrect use of the PPE, and thereby reduced the effectiveness of the PPE.

Hand hygiene practices are fundamental to preventing the spread of infection. During the COVID-19 pandemic there was an increased emphasis on the importance of hand hygiene practices and reported increases in compliance rates, with added emphasis on the necessary hand hygiene steps during the doffing process, to prevent self-contamination (28). A retrospective cohort study looking at risk factors of HCW with coronavirus disease in Wuhan, identified sub-optimal hand hygiene practices after contact with a patient with COVID-19 as a contributing factor in HCW acquiring COVID-19 from patients (29). Another study reported an increase in hand hygiene compliance rates from 57.0% pre-pandemic, to 85.2% during the pandemic (30). Hand hygiene practices of 10–20 times per day have been accepted as the frequency sufficient to trigger irritant contact dermatitis (7). In this study, approximately a third (197, 35.2%) of respondents reported an adverse reaction to hand hygiene products. The most reported adverse reactions were dryness (34.2%), followed by scaly skin (18.2%) and itch (16.3%). Similarly a study analyzing the relationship between hand hygiene activity during the COVID-19 pandemic (31) reported an increased frequency of hand hygiene related problems during the pandemic, with dryness (67.8%) most commonly reported (31). One study found that higher hand washing frequency and the application of alcohol-based hand rubs (ABHR), (in contrast to a decreased application of moisturizer), combined with the cumulative effects of increased PPE and glove use, predisposed HCWs to in the development of hand dermatitis (32).

While female HCWs have been identified as being an increased risk of skin-related adverse PPE reactions (14, 15), contributing factors to consider include the use of cosmetics by female HCWs, and differences in work patterns with females HCWs employed in roles where PPE use for extended periods of time occurred (17), for example, nurses working on a medical ward would be required to wear PPE for extended periods of time, as opposed to a Doctor in a clinic room, who can remove their PPE between patients. This is consistent with the workforce. In this study, the majority of the survey respondents were female (83.7%), with (66.0%) being nurses.

### 4.1 Limitations

As this study was completed through an online survey it was not possible to examine the participants’ self-reported pre-existing conditions or reactions to PPE in more detail. The guidelines for managing patients with COVID-19 evolved over time, and the minimum requirements for PPE use changed with these; at the time of the survey surgical masks were recommended for all staff working in both high and low risk settings which probably explains the high frequency that side-effects associated with surgical mask use were reported. As clinical staff used both surgical and N95 respirators, it was not possible to disambiguate whether the reported side effects were associated with face coverings in general or specific mask types. There is potential for a participant response bias as those who experienced an adverse reaction related to PPE use may be more likely to respond to the survey. However, when comparing the proportion of staff reporting different types of side effects the results are consistent with international studies.

## 5 Conclusion

The COVID-19 pandemic has seen a significant increase in the use of PPE. While the preventative benefits of PPE are significant, adverse events related to PPE use are frequently reported by HCWs. These findings highlight the need for innovation in PPE design to maximize protection while decreasing adverse effects with consideration of the length of time PPE is in use. Further research is warranted on establishing effective preventative strategies to decrease the incidence of PPE-related side effects. Practical applications of PPE in the workplace including education on strategies to maintain skin integrity are required.

## Data availability statement

The original contributions presented in the study are included in the article/supplementary material, further inquiries can be directed to the corresponding author.

## Ethics statement

The studies involving humans were approved by Health Service Human Research Ethics Committee. The studies were conducted in accordance with the local legislation and institutional requirements. The participants provided their written informed consent to participate in this study.

## Author contributions

KM: Data curation, Formal analysis, Project administration, Writing – review & editing. AH: Conceptualization, Formal analysis, Methodology, Supervision, Writing – review & editing. SB: Conceptualization, Formal analysis, Methodology, Project administration, Supervision, Writing – review & editing. BR: Supervision, Writing – review & editing.
