# Supplementary material for: Australian health care workers experience of PPE related side-effects. A cross-sectional survey
Source: Front Public Health. 2024 Feb 7;12:1325376. doi: 10.3389/fpubh.2024.1325376 (PMC10880185; doi:10.3389/fpubh.2024.1325376)
Supplement: Supplementary file 1 [file Table_1.docx]

Appendix Table 1. Frequency of Reported side effects from PPE use according to study site.

|  | A n = 161 n (%) | B n = 350 n (%) | C n = 48 n (%) | Total n = 559 n (%) |
| --- | --- | --- | --- | --- |
| Experienced adverse effects - goggles |  |  |  |  |
| Yes | 66 (40.9) | 59 (16.9) | 8 (16.7) | 133 (23.8) |
| Adverse effect |  |  |  |  |
| Burning / pain | 12 (7.5) | 19 (5.4) | 4 (8.3) | 35 (6.3) |
| Pressure injuries | 31 (19.3) | 22 (6.3) | 4 (8.3) | 57 (10.2) |
| Skin tear | 1 (0.6) | 5 (1.4) | 1 (2.1) | 7 (1.3) |
| Blister | 2 (1.2) | 5 (1.4) | 0 | 7 (1.3) |
| Acne | 10 (6.2) | 7 (2.0) | 1 (2.1) | 18 (3.2) |
| Abrasion | 5 (3.1) | 3 (0.9) | 0 | 8 (1.4) |
| Eczema | 3 (1.9) | 0 | 0 | 3 (0.5) |
| Allergic reaction | 2 (1.2) | 0 | 0 | 2 (0.4) |
| Blurred vision | 8 (5.0) | 7 (2.0) | 0 | 15 (2.7) |
| Fogging | 6 (3.7) | 3 (0.9) | 0 | 9 (1.6) |
| Headache / migraine | 6 (9.9) | 14 (4.0) | 2 (4.2) | 32 (5.7) |
| Others | 3 (1.9) | 2 (0.6) | 0 | 5 (0.9) |
| Experienced adverse effects - face shields | |  |  |  |
| Yes | 23 (14.3) | 28 (8.0) | 3 (6.3) | 54 (9.7) |
| Adverse effect |  |  |  |  |
| Burning / pain | 3 (1.9) | 7 (2.0) | 2 (4.2) | 12 (2.1) |
| Pressure injuries | 4 (2.5) | 10 (2.9) | 2 (4.2) | 16 (2.9) |
| Skin tear | 0 | 0 | 0 | 0 |
| Blister | 0 | 1 (0.3) | 0 | 1 (0.2) |
| Acne | 3 (1.9) | 3 (0.9) | 1 (2.1) | 7 (1.3) |
| Abrasion | 1 (0.6) | 3 (0.9) | 0 | 4 (0.7) |
| Eczema | 2 (1.2) | 3 (0.9) | 0 | 5 (0.9) |
| Allergic reaction | 2 (1.2) | 2 (0.6) | 0 | 4 (0.7) |
| Headache / migraine | 7 (4.3) | 2 (0.6) | 1 (2.1) | 10 (1.8) |
| Blurred vision | 3 (1.9) | 0 | 1 (2.1) | 4 (0.7) |
| Impaired hearing | 2 (1.2) | 1 (0.3) | 0 | 3 (0.5) |
| Impaired vision | 5 (3.1) | 5 (1.4) | 0 | 10 (1.8) |
| Fogging | 3 (1.9) | 2 (0.6) | 0 | 5 (0.9) |
| Others | 2 (1.2) | 0 | 1 (2.1) | 3 (0.5) |
| Experienced adverse effects - surgical or reusable mask | | |  |  |
| Yes | 87 (54.0) | 119 (34.0) | 15 (31.3) | 221 (39.5) |
| Adverse effect |  |  |  |  |
| Burning / Pain | 21 (13.1) | 31 (8.8) | 3 (6.3) | 55 (9.9) |
| Pressure injuries | 43 (26.7) | 51 (14.6) | 7 (14.6) | 101 (18.1) |
| Skin tear | 7 (4.3) | 5 (1.4) | 1 (2.1) | 13 (2.3) |
| Blister | 9 (5.6) | 8 (2.3) | 1 (2.1) | 18 (3.2) |
| Acne | 50 (31.1) | 62 (17.7) | 10 (20.8) | 122 (21.8) |
| Abrasion | 19 (11.8) | 21 (6.0) | 4 (8.3) | 44 (7.9) |
| Eczema | 15 (9.3) | 14 (4.0) | 1 (2.1) | 30 (5.4) |
| Allergic reaction | 15 (9.3) | 21 (6.0) | 1 (2.1) | 37 (6.6) |
| Headache / migraine | 2 (1.2) | 4 (1.1) | 1 (2.1) | 7 (1.3) |
| Asthma | 3 (1.9) | 0 | 0 | 3 (0.5) |
| Dry skin | 2 (1.2) | 1 (0.3) | 0 | 3 (0.5) |
| Others | 4 (2.5) | 12 (3.4) | 0 | 16 (2.9) |
| Location of adverse effect - surgical or reusable mask | | |  |  |
| Nose | 54 (33.6) | 54 (15.5) | 8 (16.7) | 116 (20.8) |
| Cheeks | 57 (35.4) | 61 (17.4) | 5 (10.4) | 123 (22.0) |
| Forehead | 3 (1.9) | 12 (3.4) | 0 | 15 (2.7) |
| Ear | 56 (34.8) | 83 (23.8) | 8 (16.7) | 147 (26.3) |
| Chin | 19 (11.8) | 27 (7.7) | 6 (12.5) | 52 (9.3) |
| Mouth | 0 | 6 (1.7) | 3 (6.3) | 9 (1.6) |
| Eyes | 1 (0.6) | 5 (1.4) | 0 | 6 (1.1) |
| Other | 2 (1.2) | 0 | 0 | 2 (0.4) |
| Experienced adverse effects - N95 |  |  |  |  |
| Yes | 77 (47.8) | 88 (25.1) | 5 (10.4) | 170 (30.4) |
| Adverse effect |  |  |  |  |
| Burning / pain | 28 (17.4) | 25 (7.1) | 0 | 53 (9.5) |
| Pressure injuries | 55 (34.2) | 66 (18.9) | 3 (6.3) | 124 (22.2) |
| Skin tear | 3 (1.9) | 10 (2.9) | 1 (2.1) | 14 (2.5) |
| Blister | 6 (3.7) | 9 (2.6) | 1 (2.1) | 16 (2.9) |
| Acne | 36 (22.4) | 31 (8.9) | 2 (4.2) | 69 (12.3) |
| Abrasion | 16 (9.9) | 15 (4.3) | 0 | 31 (5.5) |
| Eczema | 6 (3.7) | 5 (1.4) | 1 (2.1) | 12 (2.1) |
| Allergic reaction | 8 (5.0) | 4 (1.1) | 1 (2.1) | 13 (2.3) |
| Headache / migraine | 3 (1.9) | 3 (0.9) | 0 | 6 (1.1) |
| Asthma / Shortness of breath | 5 (3.1) | 1 (0.3) | 0 | 6 (1.1) |
| Other | 3 (1.9) | 5 (1.4) | 0 | 8 (1.4) |
| Location of adverse effect - N95 |  |  |  |  |
| Nose | 59 (36.6) | 69 (19.7) | 4 (8.3) | 132 (23.6) |
| Cheeks | 52 (32.3) | 43 (12.3.) | 1 (2.1) | 96 (17.2) |
| Forehead | 3 (1.9) | 8 (2.3) | 0 | 11 (2.0) |
| Ears | 41 (25.5) | 47 (13.4) | 2 (4.2) | 90 (16.1) |
| Chin | 13 (8.1) | 9 (2.6) | 1 (2.1) | 23 (4.1) |
| Mouth | 2 (1.2) | 5 (1.4) | 1 (2.1) | 8 (1.4) |
| Other | 7 (4.3) | 2 (0.6) | 0 | 9 (1.6) |
| Have the adverse effects experienced affected your work | | |  |  |
| Yes | 47 (29.2) | 53 (15.1) | 6 (12.5) | 106 (19.0) |
| Measures to treat / prevent adverse effect | |  |  |  |
| Yes | 64 (39.8) | 73 (20.9) | 10 (20.8) | 147 (26.3) |
| Measures taken |  |  |  |  |
| Hydrocolloid dressings | 16 (9.9) | 22 (6.3) | 0 | 38 (6.8) |
| Silicone / transparent film | 8 (5.0) | 14 (4.0) | 2 (4.2) | 24 (4.3) |
| Tension strap | 18 (11.2) | 26 (7.4) | 3 (6.3) | 47 (8.4) |
| Change in hairstyle | 28 (17.4) | 30 (8.6) | 3 (6.3) | 61 (10.9) |
| Moisturizer / creams | 26 (16.1) | 17 (4.9) | 5 (10.4) | 48 (8.6) |
| Medication | 6 (3.7) | 5 (1.4) | 0 | 11 (2.0) |
| Other | 3 (1.9) | 4 (1.1) | 0 | 7 (1.3) |
